# Community-associated methicillin-resistant *Staphylococcus aureus* infection of diabetic foot ulcers in an eastern diabetic foot center in a tertiary hospital in China: a retrospective study

**DOI:** 10.1186/s12879-023-08631-z

**Published:** 2023-10-03

**Authors:** Yixin Chen, Jie Yang, Ying Wang, Jiaxing You, Weifen Zhu, Chao Liu, Yi Luan, Lin Li, Hong Li

**Affiliations:** 1 Department of Endocrinology, Zhejiang University School of Medicine Sir Run Run Shaw Hospital, 3 East Qing Chun Road, Hangzhou, 310016 China; 2 Department of Orthopedics, Zhejiang University School of Medicine Sir Run Run Shaw Hospital, 3 East Qing Chun Road, Hangzhou, 310016 China; 3 Wound and Ostomy Care Clinic, Zhejiang University School of Medicine Sir Run Run Shaw Hospital, 3 East Qing Chun Road, Hangzhou, 310016 China; 4 Department of Cardiology, Zhejiang University School of Medicine Sir Run Run Shaw Hospital, 3 East Qing Chun Road, Hangzhou, 310016 China

**Keywords:** Community-associated methicillin-resistant *Staphylococcus aureus*, Diabetic foot ulcer, Anti-microbial susceptibility pattern

## Abstract

**Background:**

Diabetic foot concerns are a major public health problem. Methicillin-resistant *Staphylococcus aureus* (MRSA) plays a significant role in diabetic foot ulcers. Community-associated MRSA has become notorious for skin and skin soft tissue infections over the last two decades. This study investigated MRSA infection in diabetic foot patients at a tertiary hospital, focusing on the epidemiology and characteristics of community-associated MRSA.

**Methods:**

A total of 149 patients with diabetic foot infection whose culture results indicated *Staphylococcus aureus* as the source were selected. Epidemiological investigations, clinical characteristics, laboratory index records, antibiotic susceptibility analysis, and clinical outcome tracking were performed in all cases. Based on oxacillin resistance using the Vitek Compact 2 system, cases were divided into methicillin-sensitive *Staphylococcus aureus* and MRSA groups. Subgroup analysis of the MRSA group was performed in accordance with the Centers for Disease Control definition: community-associated MRSA and hospital-associated MRSA.

**Results:**

The MRSA group (n = 41, 27.5%) had a longer duration of ulcers and hospital stay and higher hospitalization costs than the methicillin-sensitive *Staphylococcus aureus* group (n = 108, 72.5%). According to the classification criteria of Infectious Diseases Society of America, the severity of infection in the community-associated MRSA group was higher than that in the hospital-associated MRSA group. The analysis of antimicrobial susceptibility of 41 MRSA isolates showed that the resistance rates to erythromycin, clindamycin, quinolone, gentamicin, tetracycline, and rifampicin were 78.0%, 68.3%, 31.7%, 17.1%, 9.8%, and 2.4%, respectively. All the MRSA strains were sensitive to linezolid, tigecycline, and vancomycin. The resistance rates to quinolones and gentamycin in the community-associated MRSA group (both 0%) were lower than those in the hospital-associated MRSA group.

**Conclusion:**

Emergence of MRSA in diabetic foot ulcer was associated with a prolonged wound duration and increased consumption of medical resources. Community-associated MRSA strains predominated among MRSA isolates from diabetic foot wounds and caused more severe infections.

## Background

*Staphylococcus aureus* (*S. aureus*) is a commensal bacteria that usually exists asymptomatically in all parts of the human body, such as the skin, skin glands, and mucous membranes, including the nose and gut of healthy people [1]. Diabetic foot ulcer is a major public health problem, which has attracted close attention worldwide, and its diagnosis [2] and treatment [3, 4] methods are constantly progressing. For decades, *S. aureus* has maintained its dominance in diabetic foot wounds [5, 6]. Diabetic foot ulcers (DFU) infected by *S. aureus* may progress to abscesses, osteomyelitis, and even gangrene, which is a common cause of hospitalization in patients with diabetes. The propensity of *S. aureus* to form biofilms complicates the treatment of bones and joints, which increases the demand for surgical debridement and amputation [7].

Methicillin-resistant *S. aureus* (MRSA) was first identified in 1961 and then became a global epidemic, with many countries reporting 50% or higher rates of MRSA infection in hospitals since the 1980s [8]. MRSA infections were limited to hospitals until healthy individuals without a connection to healthcare facilities were reported to be infected with MRSA. Community-associated MRSA (CA-MRSA) clones emerged in the late 1990s and have been rapidly spreading in hospital environments over the past few decades [9]. CA-MRSA is commonly associated with skin and soft tissue infections (SSTIs) and is highly susceptible to non-β-lactam antibiotics [10]. In most parts of Asia, including China, sequence type (ST) 59 is the predominant CA-MRSA clone, whereas the most prevalent healthcare-associated MRSA (HA-MRSA) clones are ST5 and ST239 [11, 12].

A meta-analysis showed that the prevalence of MRSA is 16.8% in patients with diabetic foot infections (DFIs) [13]. It has been reported that detection of MRSA isolates is associated with treatment failure in infected DFUs, regardless of the antibiotic agents taken [14]. However, another finding suggested that there was no difference in healing time between MRSA and methicillin-sensitive *S. aureus* (MSSA) infected ulcers after a timely surgical procedure for osteomyelitis [15]. Currently, there are few studies about the status of CA-MRSA infections in DFU in China [16].

In this study, we examined the demographic features, clinical characteristics, antimicrobial resistance patterns, and medical resource expenditure related to *S. aureus* and MRSA infections in DFU patients at a tertiary hospital.

## Materials and methods

### Study design

This retrospective study aimed to investigate MRSA infection in patients with DFU treated within a tertiary hospital in eastern China, with a particular focus on the epidemiology and characteristics of CA-MRSA infection.

### Participants

From July 1, 2018 to November 20, 2022, patients hospitalized for DFU, and associated *S. aureus* infections were included. According to the guidelines of the Infectious Diseases Society of America (IDSA) [17], diabetic foot infection was defined as the presence of at least two of the following: local swelling or induration, erythema, local tenderness or pain, local warmth, and purulent discharge. Patients were divided into two groups (MSSA and MRSA) based on the wound cultural results. The Meggitt–Wagner classification, IDSA, and International Working Group on the Diabetic Foot (IWGDF) classifications [17] were used to describe the severity of DFU.

For the MRSA group, patients were further categorized into two epidemiological classes using the Centers for Disease Control definition [18]: (1) healthcare-associated (HA) cases were classified as either community-onset (cases with a healthcare risk factor but with a culture obtained ≤ 48 h after hospital admission) or hospital-onset (cases with a culture obtained > 48 h after admission); and (2) community-associated (CA) cases were identified as community-onset without healthcare risk factors. Healthcare risk factors were documented as follows: presence of an invasive device at admission, history of MRSA infection or colonization, history of surgery, hospitalization, dialysis, or residence in a long-term care facility in the previous year. Diabetic foot infections usually occur in the community, therefore, differentiation between CA- and HA-MRSA in DFU depends on whether the patient has healthcare risk factors.

### Clinical procedure

Following the protocols recommended by the IWGDF [19], our multidisciplinary team indiscriminately provided medical care in both groups, including blood glucose regulation, perfusion improvement by prostaglandins or antiplatelet drugs, and postoperative dressings. The antibiotic therapy was first managed empirically and then modified according to the results of an antibiogram from the culture. Additionally, the MRSA-infected patients were administered linezolid or vancomycin.

Patients with Wagner 3 grade or higher usually require surgery. General, spinal, or regional anesthesia was given to the patients depending on the anesthesiologist. During the operation, nonviable and infected soft tissues and bones were excised and debrided. The edges of debridement were removed until the soft tissue and bone appeared macroscopically normal. The defect created by debridement was filled with polymethylmethacrylate premixed with gentamycin (Cemex® Genta, Tecres Spa, Verona, Italy) as a spacer.

### Data collection

The demographic and clinical characteristics of patients were collected at the time of admission. We abstracted this information using a standard clinical chart. We also evaluated whether each patient had comorbidities, such as retinopathy, nephropathy, neuropathy, peripheral arterial disease (ankle-brachial index < 0.9), atherosclerotic cardiovascular disease, or cerebrovascular disease. Routine laboratory blood tests were performed on the patients’ blood samples and results were recorded. Indices for medical economics, including hospital cost, length of hospital stay, and the number of surgical procedures, were recorded. Clinical outcomes including minor amputation (below the ankle) and major amputation (above the ankle) were noted.

### Microbiological methods and antimicrobial susceptibility testing

Specimens were obtained by tissue biopsy. Isolate species were confirmed using an automated Vitek Compact 2 system (bioMérieux, France). *S. aureus* isolates were identified as MRSA if they were resistant to oxacillin. The susceptibility of oxacillin was determined by the disk diffusion method. The minimum inhibitory concentrations of ciprofloxacin, levofloxacin, moxifloxacin, nitrofurantoin, rifampicin, tetracycline, clindamycin, gentamicin, and erythromycin were determined by the agar dilution method. Furthermore, the minimum inhibitory concentrations of vancomycin, linezolid, and tigecycline were determined by the broth microdilution method. Antimicrobial Susceptibility Testing results were interpreted in accordance with the Clinical and Laboratory Standards Institute guidelines [20].

### Statistical analysis

Quantitative data with normal distributions are presented as means ± standard deviations (SD). Data with non-normal distributions are presented as medians (25% quarxztile–75% quartiles). Continuous variables were compared using the independent samples t-test for normally-distributed data and the Mann–Whitney U test for non-normally-distributed data. Categorical variables were analyzed using the Chi-square test or Fisher’s exact test. Statistical significance was set at P < 0.05. Statistical analyses were performed using SPSS software version 26.0 (IBM Corp., Armonk, NY, USA).

### Ethical approval

This study was conducted in accordance with the Declaration of Helsinki of 1975, as revised in 2013, and approved by the Human Research Ethics Committee of Sir Run Run Shaw Hospital (Acceptance number: 2023-426-01). All participants provided written informed consent upon admission authorizing the use of their data from the clinical practice for subsequent publication.

## Results

### Baseline microbiological characteristics of diabetic foot wound culture

**Fig. 1:**
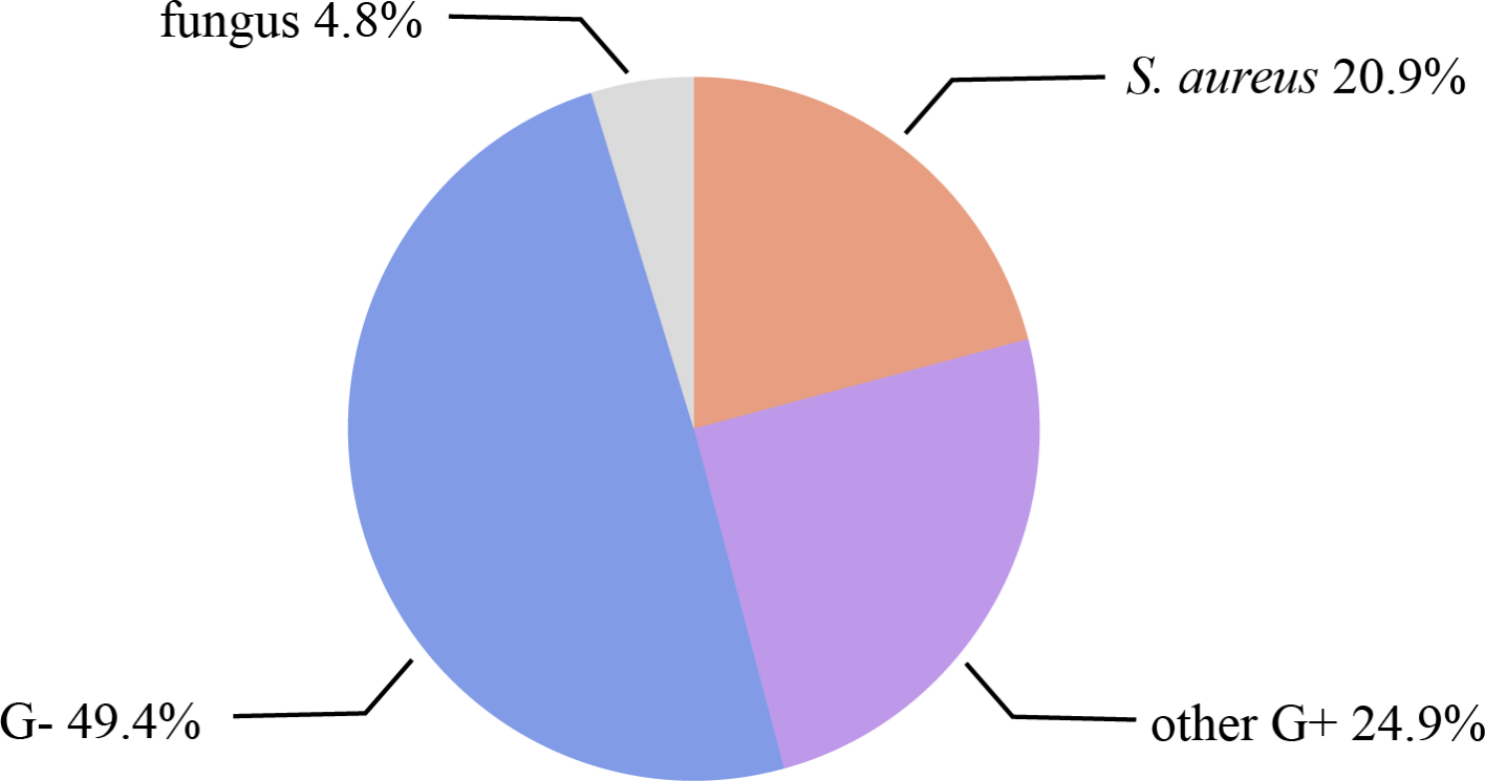
Baseline microbiological characteristics of diabetic foot wound culture from 600 patients. A total of 712 positive strains of pathogenic bacteria were isolated from 600 cases, including 326 strains (45.8%) of Gram-positive (G+) bacteria, 352 strains (49.4%) of Gram-negative (G-) bacteria and 34 strains (4.8%) were fungus. There were 149 strains of *S. aureus*, accounting for 21%, which was the highest proportion in single species

From July 2018 to December 2022, a total of 776 specimens were obtained from patients with diabetic foot ulcers in our diabetic foot center and sent to the microbiology laboratory of our hospital for testing. We did not culture anaerobes due to transportation conditions. A total of 712 positive strains of pathogenic bacteria were isolated from 600 cases, including 326 strains (45.8%) of gram-positive (G+) bacteria, 352 strains (49.4%) of gram-negative (G+) bacteria and 34 strains (4.8%) were fungus (Fig. 1). There were 149 strains of *S. aureus*, accounting for 21%, which was the highest proportion in single species.

### Demographic and clinical characteristics of participants

In total, 149 patients met the inclusion criteria out of the 600 cases tested. Among these patients, 41 *S. aureus* isolates (27.5%) were identified as MRSA and 108 isolates (72.5%) were identified as MSSA. Patients in the MRSA group had a significantly longer DFU duration (median days 60) than those in the MSSA group (median days 30, P = 0.043). For laboratory tests, patients in the MRSA group had significantly lower HbA_1c_ (median value 8.3%) than those in the MSSA group (median value 9.4%, P = 0.027). Regarding clinical outcomes, no statistical differences in minor/major amputations or surgical procedures were observed between the two groups. However, the MRSA group had a significantly longer hospital stay (median days 9) and higher hospital costs (median value 21,154 yuan) than the MSSA group (median days 7, P = 0.007; median value 18,929 yuan, P = 0.036) (Table 1).

We further analyzed the data from the CA- and HA-MRSA subgroups. Patients in the HA-MRSA group had a longer course of DM (15.73 ± 6.68 vs. 9.84 ± 7.30 years, *P* = 0.010) and a longer duration of DFU (median days 135 vs. 20, *P* = 0.002). More patients in the HA group had a history of DFU (54.5%) compared to patients in the CA group (21.1%) (*P* = 0.028). More patients (86.4%) developed nephropathy in the HA group compared to the CA group (42.1%, *P* = 0.003) (Table 1).

**Table 1:** Demographic and clinical characteristics of participants

Variable	MSSA group	MRSA group	P	CA group	HA group	P
n	108	41		19	22	
Age (years)	61.29 ± 11.57	61.44 ± 12.61	0.944	60.32 ± 11.68	62.41 ± 13.56	0.602
Male	83.3%	82.9%	0.953	78.9%	86.4%	0.831
DM duration (years)	12.5 (10–20)	12 (8.5–20)	0.688	9.84 ± 7.30	15.73 ± 6.68	**0.010**
DFU duration (days)	30 (15–60)	60 (12–195)	**0.043**	20 (10–60)	135 (39–365)	**0.002**
History of DFU	39.8%	39.0%	0.930	21.1%	54.5%	**0.028**
**Comorbidities**						
Retinopathy	38.9%	41.5%	0.774	26.3%	54.5%	0.067
Nephropathy	79.6%	70.6%	0.280	42.1%	86.4%	**0.003**
Neuropathy	92%	89%	0.773	84%	95%	0.495
PAD	29%	26%	0.681	21%	27%	0.922
ASCVD	27%	11%	0.067	11%	14%	1.000
**Laboratory test**						
HbA_1c_ (%)	9.4 (7.8–11.0)	8.3 (7.4–9.7)	**0.027**	8.2 (7.7–10.2)	8.4 (7.3–9.6)	0.889
WBC (*10^9^/L)	8.5 (6.2–11.2)	8.2 (6.1–12.1)	0.980	9.2 (7.5–13.5)	6.7 (5.3–11.4)	0.111
CRP (mg/L)	34.2 (8.4–90.1)	73.1 (10.2-134.4)	0.170	87.7 (9.5–192)	36.6 (10.4–93.5)	0.320
PCT (ng/ml)	0.07 (0.30–0.85)	0.55 (0.19–1.79)	0.173	0.97 (0.17–3.60)	0.44 (0.11–0.92)	0.386
ESR (mm/hr)	79 (44–105)	69 (41–109)	0.931	75 (35–121)	69 (47–103)	0.977
eGFR (ml/min/1.73m^2^)	84 (59–97)	72 (40–96)	0.196	88 (66–102)	56 (5–84)	**0.041**
MALB/CR (mg/g)	120 (34–687)	78 (22–226)	0.144	73 (22–342)	78 (40–200)	0.759
Albumin (g/L)	32 (28–36)	32 (28–36)	0.958	30 (25–34)	34 (29–37)	0.114
**Clinical outcome**						
Hospital costs	18,929 (12,700–24,897)	21,154 (16,065–30,620)	**0.036**	23,928(18,167–52,124)	20,127(15,604–26,001)	0.123
Hospital stays	7 (5–9)	9 (7–11)	**0.007**	10 (6–14)	8 (7–10)	0.371
Surgical procedure	1 (1–1)	1 (1–1)	0.186	1 (1–1)	1 (1–1)	0.239
Minor amputation	67.59%	59.10%	0.191	52.6%	59.1%	0.678
Major amputaion	1.85%	7.32%	0.252	15.8%	0.0%	0.182

DM: diabetes mellitus; PAD: peripheral arterial disease; ASCVD: atherosclerotic cardio/cerebro-vascular disease; HbA_1c_: glycated hemoglobin A_1c_; WBC: white blood cell; CRP: C-reative protein; PCT: procalcitonin;ESR: erythrocyte sedimentation rate; eGFR: estimated glomerular filtration rate; MALB/CR: microalbuminuria/creatinin; CA: community-associated; HA: healthcare-associated

We analyzed the Wagner and IDSA/IWGDF classifications of all patients with DFU. The proportion of IDSA grade 4 was higher (*P* = 0.009) in the MRSA group (46%) than in the MSSA group (29%). In the subgroup analysis, the CA group had more IDSA grade 4 cases (63%) than did the HA group (32%, *P* = 0.045). There was no significant between-group difference in the constituent ratio of each Wagner grade (Table 2).

**Table 2:** Wagner and IDSA/IWGDF classifications of DFU cases

	MSSA	MRSA	P	CA	HA	P
	n (%)	n (%)		n (%)	n (%)	
Wagner classification			0.541			0.623
Grade 2	10 (9)	3 (7)		1 (5)	2 (9)	
Grade 3	24 (22)	9 (22)		3 (16)	6 (27)	
Grade 4	74 (69)	28 (68)		14 (74)	14 (64)	
Grade 5	0 (0)	1 (2)		1 (5)	0 (0)	
IDSA/IWGDF classification			**0.009**			**0.045**
Grade 2	10 (9)	0 (0)		0 (0)	0 (0)	
Grade 3	67 (62)	22 (54)		7 (37)	15 (68)	
Grade 4	31 (29)	19 (46)		12 (63)	7 (32)	

CA: community-associated; HA: healthcare-associated; IDSA: Infectious Diseases Society of America; DFU: diabetic foot ulcer; IWGDF: International Working Group on the Diabetic Foot; MSSA: methicillin-sensitive *Staphylococcus aureus*; MRSA: methicillin-resistant *Staphylococcus aureus*

Forty-one MRSA strains were collected from patients with DFU. Resistance rates to penicillin and oxacillin were both 100%. Resistance rates to erythromycin (78.0%) and clindamycin (68.3%) were relatively high. Resistance rates to quinolones, gentamicin, tetracycline, and rifampicin were 31.7%, 17.1%, 9.8%, and 2.4%, respectively. None of the MRSA isolates were resistant to linezolid, tigecycline, vancomycin, nitrofurantoin, or quinupristin/dalfopristin. Additionally, in the CA-MRSA group, the resistance rates to quinolones and gentamicin were significantly lower than those in the HA-MRSA group (0% vs. 59.1%, *P* < 0.001; 0% vs. 31.8%, *P* = 0.022) (Table 3).

**Table 3:** Antimicrobial susceptibility of MRSA strains

Antibiotics	Resistance Rate			P (CA vs. HA)
	MRSA (n = 41)	CA (n = 19)	HA (n = 22)	
Oxacillin	100.0%	100.0%	100.0%	
Penicillin	100.0%	100.0%	100.0%	
Quinolones	31.7%	0.0%	59.1%	**< 0.001**
Clindamycin	68.3%	63.2%	72.7%	0.511
Erythromycin	78.0%	73.7%	81.8%	0.803
Gentamicin	17.1%	0.0%	31.8%	**0.022**
Tetracycline	9.8%	10.5%	9.1%	0.877
Nitrofurantonin	0.0%	0.0%	0.0%	
Quinupristin/Dalfopristin	0.0%	0.0%	0.0%	
Rifampicin	2.4%	0.0%	4.5%	1.000
Tigecycline	0.0%	0.0%	0.0%	
Vancomycin	0.0%	0.0%	0.0%	
Linezolid	0.0%	0.0%	0.0%	

MRSA: methicillin-resistant *Staphylococcus aureus;* CA: community-associated; HA: healthcare-associated

## Discussion

In our study, patients in the MRSA group had a significantly longer DFU duration before admission. Similar findings have been reported (i.e., that chronic unhealed wounds promote the emergence of multidrug-resistant organisms) [5, 21], which was attributed to frequent exposure to antibiotics and contact with healthcare settings. Better blood glucose control (lower HbA_1c_% value) within the MRSA group was probably due to repeated visits to the doctors for a longer duration of DFU. Our results also showed that the MRSA infections were more serious and that much more medical expenditure was incurred by the MRSA group, which manifested as markedly longer hospital stays and higher hospital costs. However, the amputation rates (including minor and major) and frequencies of surgical procedures were similar between the two groups. The additional consumption for MRSA patients may come from systemic support therapy and strengthened, prolonged antibiotic treatment, which requires linezolid or vancomycin. Studies have also shown that MRSA osteomyelitis does not predict worse prognosis but costs more in terms of healthcare resources [15, 22]. Simultaneous systematic diseases often also incur additional hospital costs and stays; however, in this study, participants with heart failure, pneumonia, or stroke attack were not common. The total number of events was less than five; therefore, it was not considered in our analysis.

To further study the clinical characteristics of MRSA cases, we conducted a subgroup analysis (CA-MRSA vs. HA-MRSA) according to epidemiological information. Patients in the HA-MRSA group had longer courses of DM and DFU. This can be explained by the fact that patients with a long course of disease have more access to medical institutions, thus increasing the chance of infection or colonization with multi-drug-resistant bacteria from hospital environments. Furthermore, a higher proportion of patients in the HA-MRSA group had a history of foot ulcer and nephropathy. The comparison of comorbidities between the two groups (Table 1) suggested that the patients in the CA-MRSA group had a better general physical condition than did those in the HA-MRSA group.

Notably, we found that the severity of infection in the CA-MRSA group was higher than that in the HA-MRSA group. The clinical manifestation suggests that CA-MRSA strains are more virulent than traditional HA-MRSA strains. Studies suggest that CA-MRSA clones have enhanced virulence and fitness compared to HA-MRSA clones, which may contribute to the epidemiological success of *S. aureus* [9]. CA-MRSA clones caused infections in the healthy individuals [23, 24], often leading to severe diseases [25, 26]. In animal infection models, CA-MRSA strains were significantly more virulent than HA-MRSA [27, 28]. Additionally, it was found that increased virulence of CA-MRSA strains was accompanied by increased viability in human neutrophils [28]. The observations suggest that CA-MRSA strains have higher virulence and ability to evade host defenses compared to conventional HA-MRSA strains. Enhanced virulence may not only increase the severity of the disease, but may also prolong the course of the disease, which would further increase the chances of pathogen transmission.

Over the past five years, several studies have investigated MRSA populations in China using the genome sequencing method, which provides a comprehensive genetic background reference [29, 30]. It was shown that ST59 was the most common MRSA ST nationwide, however ST5 was the most prevalent strain in Zhejiang Province specifically. Traditional HA-MRSA strains were mostly resistant to quinolones, whereas classic CA-MRSA ST59 clones had very low resistance rates to quinolones.

We investigated the antimicrobial susceptibility of 41 MRSA strains isolated from DFU wounds. Only MRSA strains were included in this investigation as MSSA strains were sensitive to most of the antibiotics except penicillin. Overall, these MRSA isolates exhibited relatively low resistance to quinolones and gentamycin. After these patients were subdivided into CA-MRSA and HA-MRSA groups, resistance rates to quinolones and gentamycin in the HA-MRSA group were considerably higher than those in the CA-MRSA group. An antibiotic susceptibility pattern has been used to differentiate CA-MRSA and HA-MRSA in several studies [31–33]. This method was proven to be highly consistent with the genotyped differentiation among MRSA isolates [34, 35]. Considering its convenience and affordability, antimicrobial phenotyping remains of great significance for the identification and tracking of CA-MRSA in clinical practice.

From another perspective, the antibiotic resistance characteristics of the HA-MRSA group were different from those of the traditional HA-MRSA clones. Overall, bacterial resistance was reduced. Therefore, we speculated that, in addition to HA-MRSA clones, classic CA-MRSA clones accounted for some nosocomial infections. The invasion of CA-MRSA clones into hospital environments has resulted in this change. Overall, CA-MRSA strains were predominant among MRSA-infected diabetic foot wounds. Chen and colleagues showed that, for HA-MRSA infections, the prevalence rate of ST59 strains significantly increased in 2015 at the Sir Run Run Shaw Hospital [36]. This epidemiological phenomenon has also been reported worldwide. The incidence of CA-MRSA infections is increasing, whereas the HA-MRSA infection rate is generally declining [37–39]. Reports have even suggested that CA-MRSA has nearly taken over from traditional hospital-associated MRSA (HA-MRSA) clones as a significant cause of nosocomial infections [40–43]. Isolation of CA-MRSA clones increased 10 times compared with HA-MRSA clones in San Francisco. USA300 was the most frequently isolated strain in both CA and HA infections [44]. Furthermore, mathematical models predict that CA-MRSA clones will eventually replace conventional HA-MRSA strains in hospitals [45, 46].

CA-MRSA is commonly found in patients with SSTIs. In 2012, a study reported a high prevalence of CA-MRSA infections in northern Saskatchewan in 2006. Data from eight years in this region showed that SSTIs accounted for a large proportion of CA-MRSA infections among the 2731 cases [47]. The strong relationship between CA-MRSA strains and SSTIs determines the substantial clinical significance of CA-MRSA in DFIs, which usually begin with SSTI. The gradually increasing prevalence of CA-MRSA clones has put these patients at a higher risk of colonization by MRSA for subsequent infection. Furthermore, the high pathogenicity of CA-MRSA poses a greater threat to immunocompromised patients with diabetes.

Currently, there are few studies on CA-MRSA in DFUs. In 2014, an analysis of six tigecycline clinical trials showed that, over half of MRSA-isolated patients with DFI were genetically classified as CA-MRSA strains [48]. Gabriela et al. [49] reported the first CA-MRSA USA300 clone associated with DFI in Mexico in 2015. In a Portuguese DFI study, results showed that a majority of the isolates were confirmed as CA-MRSA clones, of which the EMRSA-15 clone was the most prevalent [50]. In our study, we suspected that CA-MRSA isolates predominated in the DFU wounds in our hospital based on the antibiotic susceptibility phenotype of MRSA clones. However, further detection using genome sequencing is required to determine the genotype profile of MRSA clones in DFU.

Combined with high toxicity and drug resistance, CA-MRSA brings great challenges to the treatment of DFI. Surgical debridement remains the essential way to maximally remove the pathogen from the wound, and adequate courses of antibiotics are complementary to surgical treatment. However, increased disinfection and contact isolation in healthcare settings and education on disinfection of patients’ household environment are effective methods to prevent further transmission of CA-MRSA.

By comprehensively defining the clinical profile of *S. aureus* infections in DFU wounds in eastern China, our findings could allow for improved treatment strategies and outcomes in the future for the condition in the country. Furthermore, by speculating that CA-MRSA clones had become dominant in the nosocomial MRSA population and caused more severe infections in DFU, we have provided an increased knowledge of the condition and its effectors, highlighting the need for preventative measures. By further investigating this topic, advancements could be made to find a method to reduce the number or effect of CA-MRSA strains circulating in hospital settings, improving the patient and healthcare worker experience.

This study has some limitations. First, we obtained information from the medical records in our hospital. The size of the HA-MRSA group may have been underestimated because hospitalizations at other institutions may not have been noted. If this were the case, CA-MRSA clones would then represent an even higher proportion of nosocomial infections. Second, the study population was relatively small, which may limit the statistical conclusions regarding the true differences between the two groups. Third, we did not retain the MRSA strains cultivated from DFU wounds during patients’ hospitalization. Inadequate preparation limited further in-depth investigation of the genotyping of the MRSA isolates.

## Conclusions

Our study comprehensively describes the clinical profile of *S. aureus* infections in DFU wounds in eastern China. MRSA isolation is associated with a longer duration of diabetic foot wounds, and MRSA infections consume more medical resources. Notably, we speculated that CA-MRSA clones had become dominant in the nosocomial MRSA population and caused more severe infections in DFU. The increasing number of CA-MRSA strains circulating in hospital settings presents a challenge for patients and healthcare workers. Antimicrobial phenotyping is a simple and effective method for clinically defining CA-MRSA. Genome sequencing is a powerful tool to precisely trace the epidemiological evolution of MRSA.

## Data Availability

The data presented in this study are available on reasonable request from the corresponding author. The data are not publicly available due to privacy of the patients.

## Abbreviations

CA-MRSA: Community associated methicillin resistant *Staphylococcus aureus*
DFI: Diabetic foot infection
DFU: Diabetic foot ulcer
HA-MRSA: Healthcare associated methicillin resistant *Staphylococcus aureus*
IDSA: Infectious Diseases Society of America
IWGDF: International Working Group on the Diabetic Foot
MRSA: Methicillin resistant *Staphylococcus aureus*
MSSA: Methicillin sensitive *Staphylococcus aureus*
SSTI: Skin and soft tissue infection
MIC: Minimum Inhibitory concentration
